# Antimicrobial resistance surveillance in Ethiopia: Implementation experiences and lessons learned

**DOI:** 10.4102/ajlm.v7i2.770

**Published:** 2018-12-06

**Authors:** Rajiha A. Ibrahim, Amete M. Teshal, Surafel F. Dinku, Negga A. Abera, Abebe A. Negeri, Feven G. Desta, Eyasu T. Seyum, Adugna W. Gemeda, Wubshet M. Keficho

**Affiliations:** 1 Ethiopian Public Health Institute, Addis Ababa, Ethiopia; 2 American Society for Microbiology, Addis Ababa, Ethiopia

## Abstract

**Introduction:**

Antimicrobial resistance (AMR) poses a global threat. High levels of AMR to commonly used antibiotics have been reported in East Africa. A situation analysis of AMR in Ethiopia also indicated high resistance levels. To prevent and contain AMR, Ethiopia established a national surveillance network.

**Objectives:**

This article describes the steps taken to prioritise AMR and establish the National Antimicrobial Resistance Surveillance System in Ethiopia, as well as present the challenges and lessons learned through implementation.

**Methods:**

In April 2017, Ethiopia had developed and approved the National AMR Surveillance Plan for laboratory-based AMR surveillance. The World Health Organization recommendations and Ethiopias’s current microbiology capacity were used to prioritise organisms for reporting. The surveillance system is comprised of a network linking the national reference laboratory with surveillance sentinel sites. Roll-out of the AMR surveillance network occurred in three phases in order to ensure successful implementation.

**Results:**

Electronic capture and transmission of data, supply chain for the microbiology laboratory and communication problems were challenges observed after implementation started. Support from Ethiopian Public Health Institute focal persons for data entry, regular scheduled communication establishment and procurement of supplies by the American Society for Microbiology were some of the measures taken to address the challenges.

**Conclusion:**

Ethiopia has demonstrated that setting up AMR surveillance in lower resource settings is possible with strong leadership and stakeholder engagement.

## Introduction

Across the globe, the emergence of antimicrobial resistance (AMR) is threatening the effective and successful treatment of infectious diseases. Drug resistance proliferates due to the improper use of drugs, poor regulation of antibiotics, limited antimicrobial stewardship, poor prescribing habits and non-compliance with prescription.^1^ Mutation of bacteria genomes by different mechanisms may lead to drug-resistant strains and antimicrobial use or misuse provides a selective advantage to resistant variants. These, in turn, lead to more common treatment failure, which can result in increased morbidity and mortality, prolonged illness, premature death and other worsened clinical outcomes.^2^ Globally, it is estimated that 700 000 people die every year from drug resistance in common bacterial infections, HIV and malaria. This number is believed to be underestimated due to poor reporting and surveillance.^2^ In addition, AMR puts a financial burden on resource-limited countries.^3^ Studies from East Africa have reported high levels of AMR to commonly used antibiotics with Gram-negatives showing 50% – 100% resistance to ampicillin and trimethoprim or sulfamethoxazole (for instance in Uganda, 100% resistance in *Escherichia coli* has been observed). The extent and burden of this resistance, however, is not being monitored through established ongoing surveillance systems.^4^

In 2009, concerned about the increasing prevalence of AMR globally, the Ethiopian Drug Administration and Control Authority, in collaboration with Management Sciences for Health or Strengthening Pharmaceutical System, conducted a situation analysis, the *Antimicrobial Use, Resistance, and Containment Baseline Survey* to understand the status of resistance and trends in the use of antimicrobial drugs in Ethiopia.^5^ The survey estimated changes in resistance of a variety of pathogens, including *Streptococcus pneumoniae, Salmonella* spp. and *Staphylococcus aureus* between 1996 and 2000 in Ethiopia. *S. pneumoniae* showed an increase in resistance to erythromycin from 0% in 1996 to 19.2% in 2000. The survey also found organisms with a high level of multidrug resistance. *Shigella dysenteriae* showed an overall resistance of 31.8%, 43.8%, 81% and 89.5% to chloramphenicol, trimethoprim or sulfamethoxazole, ampicillin and tetracycline respectively.^5^ Other studies conducted across Ethiopia also indicated increasing rates of resistance in *E. coli, Shigella* spp., *Salmonella* spp. and *S. aureus* to commonly prescribed antibiotics such as ampicillin, amoxicillin, penicillin, tetracycline and trimethoprim or sulfamethoxazole.^6,7^

In addition to increased morbidity and mortality, AMR is an increasing threat to global health security with potential economic, social and political ramifications.^8^ To promote global health security as an international priority, the Global Health Security Agenda (GHSA) was launched in 2014 as an effort by nations, international organisations and civil society to accelerate progress toward a world safe and secure from infectious disease threats. The prevention and containment of AMR was identified as one of the priority initiatives under GHSA.^9^ To contain the spread of AMR and maintain the usefulness of antimicrobial agents for the future, a focus on laboratory diagnostics, surveillance, stewardship and regulation is required in countries. In particular, laboratory-based surveillance that detects resistance patterns and monitors their spread is needed to advance a country’s understanding of its AMR burden and illuminate areas to target.^1^ In response to the findings of the 2009 situation analysis, the Federal Democratic Republic of Ethiopia began prioritising efforts to detect and combat AMR in the country and in 2016, with support from the GHSA, established a national surveillance network to detect AMR. The purpose of this article is to describe the steps taken to establish the National Antimicrobial Resistance Surveillance System in Ethiopia and present the challenges and lessons learned through implementation.

## Prioritising antimicrobial resistance in Ethiopia

Following the 2009 situation analysis, Ethiopia began to focus its efforts on disseminating information about AMR to the community and establishing a national strategy and action plan to curb the rising resistance. The National Advisory Committee on Antimicrobial Resistance Prevention and Containment, a multi-disciplinary body to govern and oversee the development and implementation of the national strategy, was established in 2011 with representation from both the human and animal health sectors of the government. By August of that year, the *National Strategic Framework for Prevention and Containment of Antimicrobial Resistance* had been developed and endorsed.^10^ The AMR national proposal was also developed in 2014. However, it was not implemented due to limited resources for implementation.

In 2015, with financial and technical support from the United States Centers for Disease Control and Prevention (CDC) through the GHSA, various multi-sector partners collaborated to produce a revised 5-year *Strategy for the Prevention and Containment of Antimicrobial Resistance for Ethiopia (2015–2020)*. The updated strategy prioritised promotion of optimal use of antimicrobials in human and animal health through effective stewardship practices, and strengthening the knowledge and evidence on antimicrobial use and resistance through One Health surveillance and research.^11^

## National Antimicrobial Resistance Surveillance System

The development and implementation of the Ethiopia AMR Surveillance Plan was a national effort led by the Ethiopian Public Health Institute (EPHI) under the Federal Ministry of Health and supported by CDC, the American Society for Microbiology (ASM) as well as Ohio State University’s Global One Health initiative. In August 2016, EPHI held a 3-day workshop to kick off discussions around AMR surveillance to address the need for a ‘surveillance system that captures the emergence of resistance, trends, its spread and utilization of antimicrobial agents in different settings’. The workshop served as an opportunity to both sensitise key stakeholders about the importance of AMR and discuss and agree on priorities and methods for surveillance implementation. Staff from EPHI, stakeholders and other decision-makers used guidance materials from the World Health Organization’s (WHO) Global AMR Surveillance System (GLASS) to inform discussions and decision-making around the selection of sites, organisms and specimens to prioritise for reporting and data management methods.^12^

By April 2017, Ethiopia had developed and approved the *National AMR Surveillance Plan*.^13^ The objective of the plan is to establish a national surveillance network capable of detecting priority AMR pathogens, analysing and reporting data, characterising resistance and generating evidence to inform the implementation of targeted prevention and control programmes.^13^ The plan outlines the activities needed to implement a national AMR surveillance system, the approach for data management and reporting, and the roles and responsibilities of clinical and laboratory stakeholders.

In addition to WHO GLASS recommendations, selection of organisms and specimens to prioritise for reporting took into consideration pathogen prevalence and the current microbiology capacity of laboratories in Ethiopia. Ethiopia chose to focus on surveillance of *E. coli, Klebsiella pneumoniae*, and *S. aureus* obtained from urine and wound specimens and all carbapenem-resistant organisms, regardless of specimen type (Table 1). It is expected that, over time, as the surveillance system is strengthened, the number of pathogens and specimen types targeted will increase.

**TABLE 1:** Priority surveillance pathogens by specimen for inclusion in Ethiopia AMR surveillance.

Specimen	Basic laboratory case definition	Priority surveillance pathogens
Urine	Significant growth in urine specimen	*Escherichia coli* *Klebsiella pneumoniae*
Wound	Isolation of pathogen in the presence of pus (Gram smear shows presence of pus with associated organism)	*Staphylococcus aureus*
Other (any specimen)	Significant growth	Carbapenem-resistant: *Acinetobacter* spp *Pseudomonas aeruginosa* *Enterobactericeae*

Ethiopia’s surveillance system is structured to connect sentinel surveillance sites to the national reference laboratory at EPHI. Each surveillance site includes a laboratory that is either affiliated with or located within a hospital. Currently, the surveillance network includes a total of 16 sentinel surveillance sites located across four regions and one city administration. Roll-out of the AMR surveillance network is occurring in three phases in order to ensure successful implementation and to effectively prepare sites for sample collection, diagnostic testing, data management and reporting. In order to prioritise sites for roll-out, EPHI, with assistance from ASM, assessed laboratories across Ethiopia in late 2016. A standardised assessment tool was used to better understand the conditions and capacities of potential AMR surveillance sites.

In the first phase of implementation, 4 of the 16 sites have been targeted to participate. The national reference laboratory at EPHI is coordinating and overseeing all surveillance activities. Focal persons from EPHI have been assigned to each of the initial four sites to support implementation. Implementation began with personnel training for clinicians and laboratory staff. ASM supported EPHI to train laboratory staff from the initial four surveillance sites in basic microbiology, antibiotic susceptibility testing and data management. A model of laboratory mentorship has been put in place. The four sites have begun receiving hands-on mentorship during which experienced laboratorians from ASM and EPHI work alongside the staff from the respective surveillance sites to ensure procedures are understood, followed and refined. To ensure that quality laboratory data is generated, all sites have been enrolled in an external quality assessment programme to evaluate diagnostic and reporting ability. As the quality of specimens sent for testing also affects laboratory data quality, Ohio State University’s Global One Health initiative conducted training for clinical and laboratory staff on proper methods for clinical specimen collection. In addition, the training aimed at introducing the purpose and goals of AMR surveillance and broadly conveying the importance of stewardship, infection prevention and control in the context of AMR surveillance.

**FIGURE 1:**
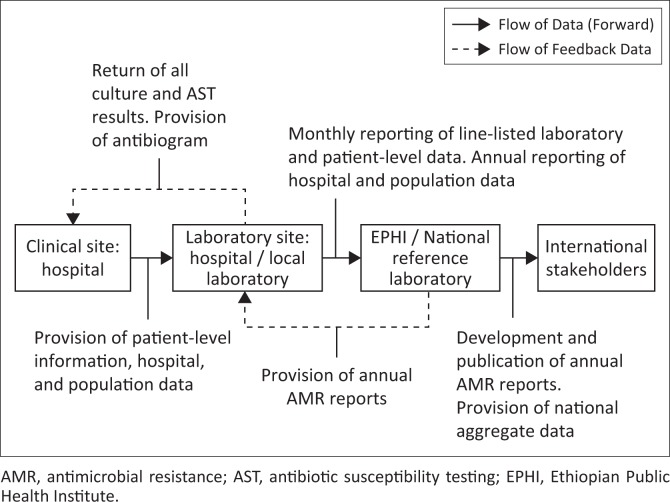
Diagram of AMR surveillance data flow within the Ethiopia National Antimicrobial Resistance Surveillance System.

The four sentinel sites receiving hands-on mentorship are currently involved in active surveillance. As part of surveillance, specimens, sent to the sentinel site laboratories, undergo routine culture and antibiotic susceptibility testing (Figure 1). Laboratory results, including measurement of the zone of inhibition, are then entered into an electronic database and sent to EPHI monthly. If a culture is positive for a priority pathogen (Table 1), the corresponding isolate will be shipped to the national reference laboratory for confirmatory testing. At EPHI, the data on pathogen prevalence and susceptibility will be analysed by using WHONET software, published and shared with the sentinel surveillance sites and regional reference laboratories, and eventually, entered into WHO GLASS.

## Challenges and lessons learned from implementation

Implementation and roll-out of AMR surveillance has started according to plan. After the fourth month of implementation, an early evaluation was conducted with support from CDC subject matter experts. The evaluation identified a number of challenges that EPHI is now taking strides to address. A primary challenge has been the integration of electronic data capture for AMR surveillance into the normal work and laboratory processes at the sentinel surveillance sites. One reason for this is that none of the sites has an established electronic laboratory information management system in use for microbiology and thus staff are not accustomed to inputting data electronically as a regular activity. As an effect of incorporating electronic entry of AMR results into their normal workflow, microbiology staff at some of the sites have reported slower turnaround times for getting laboratory results back to the ordering physicians. Frequent microbiology staff turnover at the sites has also made electronic data entry challenging, as fewer staff are available to run culture and input data.

Laboratory capacity building through on-site mentorship at the surveillance sites and provision of necessary supplies has proved a useful method for ensuring sites are producing quality data. The use of focal persons from EPHI in monitoring progress at each surveillance site has been crucial for identifying problems and supply needs, and for facilitating corrective action on laboratory practices and data reporting. Prior to the evaluation, focal persons from EPHI were visiting sites irregularly and thus creating gaps in communication. Communication between the EPHI focal persons and surveillance sites has since been improved by establishing weekly calls and arranging monthly site visits for the focal persons at EPHI to work on capacity building and quality improvement activities.

Local procurement of quality microbiology supplies is a challenge in Ethiopia. In the meantime, ASM purchased the needed AMR supplies for EPHI and the sentinel surveillance sites. For long-term sustainability, EPHI has begun working with the Pharmaceutical Fund and Supplies Agency, Ethiopia’s central procurement agency, to ensure adequate, quality supplies are available in future.

## Conclusion

Ethiopia has committed to join global partners in the detection and prevention of AMR. In a region where AMR data is under-represented and often lacking, Ethiopia has made great strides in the establishment its National Antimicrobial Resistance Surveillance System to properly understand and address the prevailing problem in the country.^14^ Ethiopia has proven that surveillance in lower resource settings is possible given strong leadership and stakeholder engagement during the planning and implementation phases of surveillance. Oversight of sentinel sites through constant communication and provision of sustainable mentorship to achieve quality data is a core element for ensuring surveillance is successful. The next steps for AMR surveillance in Ethiopia will be continuous strengthening of active surveillance sites. As capacity of the sites is strengthened, gradual engagement of additional sites will take place. This will enable the country to fulfil its goal of establishing a national AMR surveillance network that can provide quality data to further inform policies on antimicrobial prescribing and purchasing, improve the delivery of care and treatment in Ethiopia and, ultimately, reduce morbidity and mortality due to microbial infections.
