# Corrigendum: Antimicrobial resistance surveillance in Ethiopia: Implementation experiences and lessons learned

**DOI:** 10.4102/ajlm.v8i1.1109

**Published:** 2019-12-06

**Authors:** Rajiha A. Ibrahim, Amete M. Teshale, Surafel F. Dinku, Negga A. Abera, Abebe A. Negeri, Feven G. Desta, Eyasu T. Seyum, Adugna W. Gemeda, Wubshet M. Keficho

**Affiliations:** 1 Ethiopian Public Health Institute, Addis Ababa, Ethiopia; 2 American Society for Microbiology, Addis Ababa, Ethiopia

In the version of this article published earlier, the surname of the second author, Amete M. Teshale, was inadvertently misspelt as ‘Teshal’. The author’s surname should have appeared as ‘Teshale’ throughout the author list and ‘how to cite’ information section. This correction does not alter the study’s findings of significance or overall interpretation of the study results. The authors apologise for any inconvenience caused.

